# Birth asphyxia outcomes and associated factors among newborns admitted to a tertiary hospital in Eastern Uganda: A prospective cohort study

**DOI:** 10.1186/s12884-025-07603-2

**Published:** 2025-04-24

**Authors:** Grace Apio, Scovia Nalugo Mbalinda, Jimmy Patrick Alunyo, Ambrose Okibure, Brian Tonny Makoko, Molly McVoy, Elizabeth Ayebare

**Affiliations:** 1 Department of Nursing, Makerere University, Kampala, Uganda; 2 Mbale Clinical Research Institute, Mbale, Uganda; 3 Department of Public Health, Busitema University Faculty of Health Sciences, Mbale, Uganda; 4 Department of Biostatistics, School of Public Health, Makerere University, Kampala, Uganda; 5 Case Western Reserve University School of Medicine, Cleveland, USA; 6 Department of Child Psychiatry, University Hospitals Cleveland Medical Center, Ohio, USA

**Keywords:** Birth asphyxia, Hypoxic ischemic encephalopathy, Newborn and factors

## Abstract

**Background:**

Birth asphyxia (BA) is a significant global health challenge, contributing to an estimated 23% of neonatal deaths worldwide and a substantial burden of long-term disabilities. It results from interrupted blood flow and gas exchange to the fetus, leading to neuronal injury and short or long-term outcomes. While most affected newborns recover fully, a notable proportion develop hypoxic-ischemic encephalopathy (HIE), associated with high morbidity and mortality. This study aimed to describe Birth asphyxia outcomes and associated factors among newborns admitted at Mbale Regional Referral Hospital (MRRH.)

**Methodology:**

We conducted a longitudinal prospective study involving mother-baby pairs with birth asphyxia within the first 24 hour (of life admitted to MRRH. Participants were recruited using a consecutive sampling approach. Data was collected using structured questionnaires and analysed with STATA version 15. Logistic regression was employed to determine factors associated with poor outcomes among newborns with birth asphyxia, with results presented as crude and adjusted odds ratios (AOR).

**Results:**

A total of 286 mother-baby pairs participated in the study. Hypoxic ischemic encephalopathy (HIE) was observed in 70.3% of cases at admission, which decreased to 45.1% at 12 h and 24.6% at 24 h. Mortality rates were 4.6%, 4.4%, and 1.2% at admission, 12 h and 24 h, respectively. Key independent factors associated with severe HIE included referral from lower-level health facilities (AOR 4.2; CI 1.7–10.0; *P* < 0.001), passage of meconium-stained amniotic fluid (AOR 2.2; CI 1.2–4.1; *P* = 0.014), and newborn resuscitation (AOR 5.1; CI 1.8–15.0; *P* = 0.003).

**Conclusion:**

The incidence of mortality and HIE among asphyxiated newborns remains high. Referral from lower-level health facilities, the passage of meconium-stained amniotic fluid, and the need for newborn resuscitation were significant predictors of severe HIE and mortality. Strengthening maternal and neonatal care at peripheral health facilities and timely referrals could mitigate these outcomes.

**Supplementary Information:**

The online version contains supplementary material available at 10.1186/s12884-025-07603-2.

## Background

World Health Organization (WHO) defines Birth asphyxia (BA) as failure of the neonate to initiate and sustain breathing at birth or an Apgar score of less than 7 at 1 min [1]. This is due to the temporary interruption of oxygen availability that indicates a risky metabolic acidosis, even when the insult does not lead to a fatal outcome [2]. Birth asphyxia results in hypoxemia (lack of oxygen) and hypercapnia (accumulation of carbon dioxide). About 10% of these newborns will require support to adapt to the extra uterine environment and establish regular respiration [3]. Birth asphyxia is diagnosed when the APGAR (Appearance/Color, Pulse/Heart rate, Grimaces/Reflexes, Activity/Muscle tone, and Respiration) score is less than seven at 1 min by two levels, which is given a score of 0, 1, or 2 accordingly (WHO, 2018).

Globally, birth asphyxia occurs in 2 to 10 per 1000 term newborns [4], and 4 million neonatal deaths are reported to occur yearly due to the same [5]. For the last 20 years, birth asphyxia has been the leading cause of neonatal mortality, with about 28% of neonatal deaths dying annually [6]. Furthermore, the prevalence of birth asphyxia varies across the globe and Africa contributes nearly 50% of the total death [7].

In Sub-Saharan Africa, around 338,000 under-five deaths occur due to birth asphyxia [7]. Ending preventable neonatal mortality rates is a global priority under the Sustainable Development Goal (SDG) 3 agenda, and Uganda, being a member is determined to work towards achieving this goal [8].

In Uganda, birth asphyxia has been the leading cause of perinatal death at an average of 48% [9]. The country is among the first 10 countries with high neonatal mortality of 27 deaths per 1,000 live births [10]. This Neonatal mortality is higher than for Kenya, which is in the same region having 21 per 1000 live births. Birth asphyxia is associated with life-threatening complications such as Hypoxic Ischemic Encephalopathy (HIE), hypoxemia and organ failure hence death [11]. HIE carries high case fatality rates ranging between 10 and 60%, with 25% of survivors having adverse long-term neurodevelopment outcomes on the babies discharged home [12]. It further leads to numerous complications, such as post-traumatic stress disorders, low cognitive functions and neurological outcomes [13].

The government of Uganda has put different measures to reduce neonatal mortality and morbidity in the country [14]. At the national level, the Ministry of Health (MOH) adopted and recommended a number of guidelines for use in the hospitals to guide practice. Guidelines such as MPDSR tools, Essential Maternal and Newborn Clinical Care Guidelines for Uganda [15] and Uganda Clinical Guidelines [16], and others have been disseminated to all hospitals. Furthermore, different preventive measures such as improving on the three delays, proper monitoring of labour with a partograph, provision of obstetric emergency care at all levels, and timely and adequate resuscitation of Newborns with birth asphyxia to improve outcome [2].

At the hospital level, strategies such as mentorship, coaching, and training have been implemented using protocols from these guidelines to improve care and reduce birth asphyxia. However, the MPDSR report for 2020/2021 highlighted that the Bugisu region has a higher number of neonatal deaths due to birth asphyxia, with an average of 55.6%. This underscores the need for further investigation in Eastern Uganda, where the Bugisu subregion is located. Therefore, this study aimed to describe the immediate outcomes of birth asphyxia and identify factors associated with adverse outcomes among newborns admitted to Mbale Regional Referral Hospital within the first 24 h of life.

## Methods

### Study design

We employed a prospective cohort longitudinal study among 286 newborn mother pairs admitted with birth asphyxia to the Special Care Unit (SCU) at Mbale Regional Referral Hospital (MRRH) from September 1, 2022, to October 30, 2022. This study design was suitable as it allowed the researcher to monitor the newborns and track changes in the outcome variables [17].

### Study setting

Mbale Regional Referral Hospital (MRRH) is located in Mbale City, Eastern Uganda. It is a government-owned facility that serves approximately 16 districts in the eastern region of the country. The hospital has a total bed capacity of 800, with the maternity ward admitting around 8,753 pregnant women annually and a total of nearly 8,000 deliveries during the financial year 2020/2021.

According to the 2020–2021 Maternal and Perinatal Death Surveillance and Response (MPDSR) report, the Bugisu region, where MRRH is located, had the highest neonatal death rate in the country at 55.6%, compared to the other 14 regions in Uganda [9]. Initially, the hospital operated a high-dependency unit for newborns until the arrival of a neonatologist from the United States in 2012. This support led to the establishment of a Special Care Unit (SCU) to care for small and sick newborns in the region.

The study was conducted in the SCU, which has a bed capacity of 20, including 10 larger beds, 5 incubators, and 10 radiant warmers. The unit is staffed by 20 personnel, including 8 nurses, 4 midwives, 2 clinical officers, 3 medical doctors, 2 paediatricians, and 1 neonatologist. A variety of diagnostic and treatment services are provided daily, with approximately 20 sick newborns admitted to the unit from both the hospital’s labour suite and outside facilities. On average, 600 newborns are admitted to the unit each month, and approximately 150 newborns are admitted due to birth asphyxia, according to the first quarter report for 2020/2021.

### Clinical care given to newborns during the study period in SCU

Throughout study, care provided to newborns in SCU included thermoregulation via overhead warmers, incubators, and kangaroo mother care, intravenous hydration, nasogastric tubes for feeding, breastfeeding, phototherapy, antibiotics, anticonvulsants, blood transfusion, basic laboratory services including complete blood count and electrolytes, continuous positive airway pressure (CPAP) therapy [18] and intermittent as well as continuous pulse oximetry as needed. Mechanical ventilation, blood culture. Close monitoring of patients included care by nurse’s continuous physician coverage was available as needed and rounds were led daily by a neonatologist or medical officers.

### Study population

The study population for this study were all newborns/mother pairs admitted due to birth asphyxia to the neonatal intensive care unit of Mbale Regional Referral Hospital during the study period.

### Selection criteria

#### Inclusion criteria

Newborn/mother pairs admitted to the SCU with birth asphyxia, either delivered by cesarean section (CS) or spontaneous vaginal delivery (SVD), were included in the study. The newborns were either born within the hospital labour suite or referred from outside facilities, provided they were less than 24 h old at the time of admission. Only those whose mothers consented to participate in the study were included.

#### Exclusion criteria

We excluded Newborns with critically ill mothers, such as those with eclampsia who were unable to respond to questions, and were not invited to participate in the study, as obtaining consent would have been difficult. Additionally, newborns with the following conditions were excluded: (1) gross congenital malformations, (2) those whose mothers died immediately after birth, and (3) those abandoned immediately after birth.

### Study variables

The dependent variable in this study was the immediate outcomes of newborns with birth asphyxia, with the primary outcome being Hypoxic-Ischemic Encephalopathy (HIE), and secondary outcomes including hypoxemia, discharge, or mortality.

The independent variables included:

Maternal sociodemographic characteristics: age, weight, height, marital status, employment status, educational level, and parity.

Newborn characteristics: sex, gestational age, and birth weight.

Obstetric characteristics: rupture of membranes before the onset of labour, preterm birth, prolonged labour, history of antepartum haemorrhage (APH), pre-rupture of membranes, preeclampsia and eclampsia, obstructed labour, antenatal attendance, mode of delivery, and multiple births.

### Sample size determination

The Kish-Leslie formula (Kish 1965) was used to calculate the sample size required to estimate the proportion of neonates with HIE at admission, 12 and 24 h [19], given by

$$\:n=\frac{{za}^{2}p(1-p)}{{d}^{2}}$$

Where-:

n - The required sample size.

z$\:a$ - The standard z values correspond to a 95% confidence interval, which is, set at 1.96.

p - The proportion of HIE in the target population is estimated to be 24.8% as found by a study done in Mulago Hospital [20],.

d = acceptable error of 0.05.

So z$$\:a=$$1.96, *p*=0.084, and d=0.05.

n= (1.96^2^ × 0.248 × 0.752) / 0.05^2^.

*n* = 0.716/0.0025.

*n* = 286 participants.

### Sampling procedure

We used consecutive sampling to recruit eligible mother newborn pairs. Trained research assistants and the principal investigator were present in the ward 24 h a day throughout the study period to ensure uninterrupted screening and recruitment. Mothers were approached for individual consent upon arrival from referral points, as the daily incidence of birth asphyxia was unpredictable.

Hypoxic-ischemic encephalopathy (HIE) was assessed using the Thompson score, and newborn oxygen saturation was measured with a pulse oximeter. Data collection was conducted over four weeks (September–October 2022), following ethical approval. Research assistants, trained in standardized data collection procedures, performed daily assessments, including on weekends and public holidays. After obtaining maternal consent at admission, newborn parameters were recorded at birth, 12 h, and 24 h.

### Data collection instrument

Data were collected using a semi-structured, researcher-administered questionnaire comprising five sections: (1) maternal sociodemographic characteristics, (2) intrapartum conditions, (3) fetal characteristics, (4) immediate newborn care, and (5) neonatal outcomes, specifically birth asphyxia. The survey tool was developed for this study, incorporating the Thompson score for clinical assessment of hypoxic-ischemic encephalopathy (HIE). See Table 1.

To ensure linguistic and conceptual consistency, the questionnaire was translated into Lumasaba and back translated into English. Research assistants underwent standardized training on the Thompson scoring system, and the questionnaire was pretested before data collection.

**Table 1:** Thompson score evaluation tool by Biselele, Naulaers, & Tady, 2014

SIGNS	SCORES			
	0	1	2	3
Tone	Normal	Hyper	Hypo	Flaccid
Level of Consciousness	Normal	Hyperalert, Stare	Lethargic	Comatose
Fits	None	Infrequent, < 2 per hour	Frequent, > 3 per hour	
Posture	Normal	Fisting, Cycling	Strong distal flexion	Decerebrate
Moto Reflex	Normal	Partial	Absent	
Grasping	Normal	Poor	Absent	
Sucking	Normal	Poor	Absen t ± bites	
Breathing	Normal	Hyperventilation	Brief apnea	IPPV (apnea)
Fontanelle	Normal	Full, not tense	Tense	
Date and Time Total				

In this study, all newborns who scored from 1–10 were reported as 0 whereas those with ≥ 11 score were reported as 1. Where 0 represented newborn without HIE, and 1 represented newborn with HIE

In addition, the questionnaire was pretested before data collection.

### Data collection tools and procedures

Data were collected using paper-based questionnaires administered at admission, 12 h, and 24 h post-birth in English and Lumasaba. The questionnaire covered maternal sociodemographic characteristics, intrapartum events, newborn characteristics, immediate newborn care, Apgar scores at birth, and immediate outcomes. Maternal sociodemographic data were obtained through direct interviews, while information on intrapartum events, newborn characteristics, immediate newborn care, and Apgar scores was abstracted from maternity records. Immediate newborn outcomes were assessed using pulse oximetry. Discharge dates and mortality outcomes were abstracted from hospital records. Interviews were conducted in a private room, lasting 15–20 min. Data collection was conducted over four weeks (September–October 2022).

### Data management

The completed questionnaires were cross-checked immediately for completeness. Coded responses were entered into the computer using Epi Data software. The data were then transferred to STATA version 17 for analysis. Electronic data was securely backed up on a password-protected computer accessible only to the researcher. All questionnaires and consent forms were stored in a locked cabinet with access restricted to the researcher.

### Data quality control/assurance

The structured questionnaire was professionally translated from English to Lumasaba and pre-tested on mother-newborn pairs at Atutur Hospital to assess reliability, consistency, and validity. Necessary revisions were made before data collection. Research assistants received training on the study protocol to ensure standardized data collection. They were closely supervised, and completed questionnaires were reviewed after each interview to check for accuracy and completeness, allowing immediate corrections when needed.

Data was cleaned and stored daily by the principal investigator. Double data entry was conducted to identify errors, with backups maintained on a secure hard drive. All questionnaires were securely stored throughout the study.

### Data analysis

Descriptive statistics were used to summarise the data. Univariate analysis was performed to summarise the mothers’ sociodemographic factors and examine each variable individually.

Bivariate analysis using the Chi-square test was applied to determine the relationship between maternal sociodemographic characteristics and the development of HIE.

Multinomial logistic regression models were used to further analyse factors that were statistically significant at the bivariate level, as well as those strongly associated with the outcome variables (e.g., referral from lower facilities, delayed breastfeeding, meconium-stained amniotic fluid, and resuscitation with a bag and mask). This analysis aimed to control confounders by adding one variable at a time until a stable model was achieved. HIE was coded as “yes,” while no HIE was coded as “no.” Factors with an adjusted odds ratio greater than one and a p-value of less than 0.05 were considered statistically significant. A binary logistic regression model was used for mortality to determine which group was at risk.

## Results

### Socio-demographic characteristics

We interviewed 286 mothers of newborns with birth asphyxia (BA) admitted to the Special Care Unit (SCU). The majority of mothers (67.8%, 194/286) were aged between 20 and 34 years. Most were married (82.9%, 237/286), and 52.4% (150/286) had attained secondary education. Regarding employment, 78.3% (224/286) were not employed, and 58.0% (166/286) had delivered more than one child. Detailed information is provided in Table 2 below.

**Table 2:** Maternal Socio-Demographic characteristics of newborns with birth asphyxia

Variable	Frequency *n* = 286(%)	Percentage (%)
Mother’s age (years)		
15–17	39	13.6
18–34	222	77.6
> 34	25	8.7
Marital status		
Married	237	82.9
Single	49	17.1
Highest level of education		
Non formal Education	6	2.1
Secondary	150	52.4
Tertiary	19	6.6
Primary	111	38.8
Employment status		
Employed	62	21.7
Not employed	224	78.3
Number of children delivered		
1	120	42.0
> 1	166	58.0

### Newborn characteristics and immediate care given

Of the 286 newborns, 158 (55.3%) were male and 128 (44.7%) were female. The majority, 264 (93.7%), were born at term (gestational age > 37 weeks), 164 (57.3%) had meconium-stained amniotic fluid, and 260 (90.9%) were delivered in a vertex presentation.

At admission to the Special Care Unit (SCU), 265 (92.6%) of the newborns required resuscitation using a bag and mask, 36 (12.5%) were initiated on breastfeeding, and 59.9% received fluid resuscitation. Additionally, 107 (37.4%) were referred from the peripheral facilities. Detailed information is presented in Table 3.

**Table 3:** Characteristics of newborns with birth asphyxia and immediate care given at the NCU

Variables	Frequency, *n* = 286	Percentage (%)
New-born Sex		
Female	128	44.7
Male	158	55.3
Gestational age (weeks)		
< 37	22	7.7
≥ 37	264	92.3
Colour of the meconium-stained amniotic fluid		
Clear	122	43.7
Stained	164	57.3
Fetal presentation		
Breech	30	10.3
Vertex	256	89.7
Referral status of newborn		
Newborns referred from outside facilities	103	36.4
Born within the health facility	183	63.9
Immediate newborn care		
The Newborn resuscitated with a bag and mask	261	91.3
Newborns not resuscitated with bag and mask	25	8.7
Newborns initiated breastfeeding	32	11.3
Not initiated to breastfeeding	254	88.7
Newborn received any fluids resuscitation	169	59.3
Not received any fluid resuscitation	117	40.7

### Immediate outcomes of newborns with birth asphyxia at admission, 12 h and 24 h

Immediate outcomes of the newborn were defined as newborn oxygen circulation (spo2), discharged or still hospitalized, died or alive and developed hypoxic encephalopathy or not within the first 24 h of admission. Of 286 newborns with birth asphyxia enrolled in the study, 198/286 (70.3%), 119/286 (45.1%) and 60/286 (24.6%) developed hypoxic ischemic encephalopathy (HIE) at admission, 12 h and 24 h respectively (Table 4**).**

The oxygen saturation of neonates improved over time from 6/286 (2.3%) of babies with SPO2 ≥ 95% at admission to 82/268(30.6%) at 12 h and 165/244 (67.6%) at 24 h (Table 3).

The mortality rate reduced over time from 13/286(4.6%) at admission to 12(4.4%) at 12 h and 3/244 (1.2%) at 24 h. The number of babies discharged back home increased over time from 5/286 (1.7%) at admission to 8/268(3.0%) at 12 h and 65/244(26.6%) at 24 hours’ details are in Table 4.

**Table 4:** Immediate outcomes of newborns with asphyxia at admission, 12 h and 24 h

Variable	At Admission *n* = 286(%)	At 12 h *n* = 268(%)	At 24 h *n* = 244(%)
Baby developed HIE			
No (1–10)	88(31.2)	145(54.9)	184(75.4)
Yes (≥ 11)	198(70.3)	119(45.1)	60(24.6)
SPO2 (%)			
< 95	280(97.9)	186(69.4)	79(32.4)
≥ 95	6(2.3)	82(30.6)	165(67.6)
Discharged			
Yes	5(1.7)	8(3.0)	65(26.6)
No	281(98.3)	260(97.0)	175(71.7)
Status of the baby			
Died	13(4.6)	12(4.6)	3(1.7)
Alive			

### Demographic characteristics of mothers who delivered newborns with BA

At bivariate analysis, newborns of mothers aged 15–19 years were more likely to have severe HIE scores (> 10) at admission (78.8%) compared to those born to mothers aged > 34 years (48.0%). However, this association weakened by 12 h (*p* = 0.053) and was no longer significant by 24 h (*p* = 0.648), indicating that the initial influence of maternal age on HIE scores may diminish over time.

Marital status was also associated with HIE severity at admission (*p* = 0.046). Married mothers were more likely to have newborns with milder HIE scores (1–10) at admission (33.3%) compared to single mothers (18.8%). However, this relationship did not persist at 12 h (*p* = 0.312) or 24 h (*p* = 0.257).

Other maternal factors, including education level, employment status, and parity, did not show significant associations with HIE severity at any time point, as indicated by consistently high p-values (> 0.05). See Table 5.

**Table 5:** Maternal characteristics versus HIE diagnosis for asphyxiated newborns at different time points

Variable	At admission				At 12 h					At 24 h			
	Total *n* = 282(%)	HIE Score		*P*-value	Total *n* = 264(%)	HIE Score			*P*-value	Total *n* = 244(%)	HIE Score		*P*-value
		1–10 *n* = 87(%)	> 10 *n* = 195(%)			1–10 *n* = 145(%)		> 10 *n* = 119(%)			1–10 *n* = 184(%)	> 10 *n* = 60(%)	
Mothers age (age in years)				0.017					0.053				0.648
15–19	66(23.4)	14(16.1)	52(26.7)		53(20.1)	23(15.9)		30(25.2)		51(20.9)	37(20.1)	14(23.3)	
20–34	191(67.7)	60(69.0)	131(67.2)		186(70.5)	104(71.7)		82(68.9)		170(69.7)	128(69.6)	42(70.0)	
> 34	25(8.9)	13(14.9)	12(6.2)		25(9.5)	18(12.4)		7(5.9)		23(9.4)	19(10.3)	4(6.7)	
Marital status				0.046					0.312				0.257
Married	234(83.0)	78(89.7)	156(80.0)		226(85.6)	127(87.6)		99(83.2)		210(86.1)	161(87.5)	49(81.7)	
Single	48(17.0)	9(10.3)	39(20.0)		38(14.4)	18(12.4)		20(16.8)		34(13.9)	23(12.5)	11(18.3)	
Highest level of education				0.615					0.424				0.157
Non formal	6(2.1)	3(3.4)	3(1.5)		6(2.3)	5(3.4)		1(0.8)		6(2.5)	6(3.3)	0(0.0)	
Secondary	148(52.5)	43(49.4)	105(53.8)		143(54.2)	78(53.8)		65(54.6)		129(52.9)	91(49.5)	38(63.3)	
Tertiary	18 (6.4)	7(8.0)	11(5.6)		19(7.2)	12(8.3)		7(5.9)		19(7.8)	14(7.6)	5(8.3)	
Primary	110(39.0)	34(39.1)	76(39.0)		96(36.4)	50(34.5)		46(38.7)		90(36.9)	73(39.7)	17(28.3)	
Employment status				0.569					0.989				0.544
Employed	59(20.9)	20(23.0)	39(20.0)		60(22.7)	33(22.8)		27(22.7)		58(23.8)	42(22.8)	16(26.7)	
Not employed	223(79.1)	67(77.0)	156(80.0)		204(77.3)	112(77.2)		92(77.3)		186(76.2)	142(77.2)	44(73.3)	
Number of children have delivered				0.714					0.756				0.689
1	118(41.8)	35(40.2)	83(42.6)		107(40.5)	60(41.4)		47(39.5)		103(42.2)	79(42.9)	24(40.0)	
> 1	164(58.2)	52(59.8)	112(57.4)		157(59.5)	85(58.6)		72(60.5)		141(57.8)	105(57.1)	36(60.0)	

### Bivariate results of factors associated with HIE severity at different time points

Table 6 presents the bivariate result of factors Associated with HIE Severity at Different Time Points. At admission, delivery before term was significantly associated with higher HIE severity scores (HIE > 10) compared to term deliveries (16.1% vs. 6.7%, *p* = 0.013). However, this association diminished by 12 h (11.0% vs. 7.6%, *p* = 0.338) and 24 h (10.3% vs. 8.3%, *p* = 0.653).

Hypertension in pregnancy was associated with higher HIE severity scores at both admissions (25.3% vs. 11.3%, *p* = 0.003) and 12 h (20.7% vs. 10.1%, *p* = 0.019). By 24 h, the association was no longer significant (17.4% vs. 11.7%, *p* = 0.293).

Obstructed labour was significantly associated with higher HIE severity scores at all time points. At admission, 56.9% of newborns with severe HIE experienced obstructed labour compared to 43.1% with mild HIE (*p* = 0.001). Similarly, at 12 h, the proportion remained high among those with severe HIE (57.1% vs. 42.9%, *p* = 0.011), and at 24 h, this trend persisted (70.0% vs. 30.0%, *p* = 0.001), as seen in Table 6.

**Table 6:** Association between maternal intrapartum condition and HIE among newborns with BA

Variable	At admission				At 12 h				At 24 h			
	Total *n* = 282(%)	HIE Score		*P*-value	Total *n* = 264(%)	HIE Score		*P*-value	Total *n* = 244(%)	HIE Score		*P*-value
		1–10 *n* = 87(%)	> 10 *n* = 195(%)			1–10 *n* = 145(%)	> 10 *n* = 119(%)			1–10 *n* = 184(%)	> 10 *n* = 60(%)	
Delivered twins				0.647				0.714				0.147
No	250(88.7)	76(87.4)	174(89.2)		235(89.0)	130(89.7)	105(88.2)		220(90.2)	163(88.6)	57(95.0)	
Yes	32(11.3)	11(12.6)	21(10.8)		29(11.0)	15(10.3)	14(11.8)		24(9.8)	21(11.4)	3(5.0)	
Membranes rupture before the onset of labor				0.337				0.355				0.568
No	199(70.6)	58(66.7)	141(72.3)		190(72.0)	101(69.7)	89(74.8)		176(72.1)	131(71.2)	45(75.0)	
Yes	83(29.4)	29(33.3)	54(27.7)		74(28.0)	44(30.3)	30(25.2)		68(27.9)	53(28.8)	15(25.0)	
Delivered before term				0.013				0.338				0.653
No	255(90.4)	73(83.9)	182(93.3)		239(90.5)	129(89.0)	110(92.4)		220(90.2)	165(89.7)	55(91.7)	
Yes	27(9.6)	14(16.1)	13(6.7)		25(9.5)	16(11.0)	9(7.6)		24(9.8)	19(10.3)	5(8.3)	
Mode of delivery				0.422				0.767				0.183
C/S	130(46.1)	37(42.5)	93(47.7)		118(44.7)	66(45.5)	52(43.7)		112(45.9)	80(43.5)	32(53.3)	
SVD	152(53.9)	50(57.5)	102(52.3)		146(55.3)	79(54.5)	67(56.3)		132(54.1)	104(56.5)	28(46.7)	
Hypertension in pregnancy				0.003				0.019				0.293
No	238(84.4)	65(74.7)	173(88.7)		222(84.1)	115(79.3)	107(89.9)		205(84.0)	152(82.6)	53(88.3)	
Yes	44(15.6)	22(25.3)	22(11.3)		42(15.9)	30(20.7)	12(10.1)		39(16.0)	32(17.4)	7(11.7)	
Labour Obstructed				0.001				0.011				0.001
No	142(50.4)	58(66.7)	84(43.1)		136(51.5)	85(58.6)	51(42.9)		124(50.8)	106(57.6)	18(30.0)	
Yes	140(49.6)	29(33.3)	111(56.9)		128(48.5)	60(41.4)	68(57.1)		120(49.2)	78(42.4)	42(70.0)	
Bleeding before delivery				0.788				0.709				0.604
No	211(74.8)	66(75.9)	145(74.4)		199(75.4)	108(74.5)	91(76.5)		185(75.8)	141(76.6)	44(73.3)	
Yes	71(25.2)	21(24.1)	50(25.6)		65(24.6)	37(25.5)	28(23.5)		59(24.2)	43(23.4)	16(26.7)	
Attended ANC				0.178				0.842				0.985
No	4(1.4)	0(0.0)	4(2.1)		4(1.5)	2(1.4)	2(1.7)		4(1.6)	3(1.6)	1(1.7)	
Yes	278(98.6)	87(100)	191(97.9)		260(98.5)	143(98.6)	117(98.3)		240(98.4)	181(98.4)	59(98.3)	
Number of times attend ANC)				0.595				0.346				0.431
< 4	91(32.3)	30(34.5)	61(31.3)		81(30.7)	48(33.1)	33(27.7)		75(30.7)	59(32.1)	16(26.7)	
>=4	191(67.7)	57(65.5)	134(68.7)		183(69.3)	97(66.9)	86(72.3)		169(69.3)	125(67.9)	44(73.3)	
Place of delivery				0.117				0.519				0.207
At home	1(0.4)	0(0.0)	1(0.5)		1(0.4)	0(0.0)	1(0.8)		1(0.4)	0(0.0)	1(1.7)	
Hospital	273(96.8)	82(94.3)	191(97.9)		255(96.6)	141(97.2)	114(95.8)		236(96.7)	179(97.3)	57(95.0)	
On my way to hospital	8(2.8)	5(5.7)	3(1.5)		8(3.0)	4(2.8)	4(3.4)		7(2.9)	5(2.7)	2(3.3)	

### Newborn characteristics and immediate care given to newborns with BA in the first 24 h of admission to SCU

Bivariate analysis by cross-tabulation of newborn characteristics (newborn sex, gestational age, fetal presentation, and meconium-stained amniotic fluid) and immediate care that was given to the newborn at admission (newborn referral, resuscitation using bag and mask and initiation to breastfeeding) against HIE outcomes at admission, 12 and 24 h revealed the following.

At admission, a majority of the newborns that developed HIE were delivered at more than 37 + weeks of gestation (94.9% vs.86.2%, *p* < 0.012), had meconium-stained amniotic fluid (65.1% vs. 37.9%, *p* < 0.001), breech delivery (11.3% vs.4.6%, *p* < 0.073), and referrals from peripheral facilities (42.6% vs.23.0%, *p* < 0.002).

Also, newborns who received resuscitation from the referral facility developed HIE at admission than those not resuscitated (96.9% vs.82.8%, *p* < 0.001). The ones who initiated breastfeeding immediately improved compared to those not initiated (17.3% vs. 11.3%, *p* < 0.037).

At 12 h of monitoring, still-newborns delivered at more than 37 + weeks of gestation had HIE (95.8% vs. 89.0%, *p* < 0.041), newborns with meconium-stained amniotic fluid mostly reported with HIE (73.1% vs., 40.0%, *p* < 0.001), those resuscitated from referral site (95.8% vs. 88.3%, *p* < 0.023) and those that were referred from lower peripheral health facilities (45.4% vs.26.9%, *p* < 0.002) were reported to have HIE at 12 h.

At 24 h, a majority of newborns reported with meconium-stained amniotic fluid developed HIE compared to those that did not (76.7% vs.47.8%, *p* < 0.001). Furthermore, newborns referred from lower health facilities reported a higher percentage of HIE than those born from within (53.3% vs. 26.6%, *p* < 0.001), respectively at 95% confidence intervals. There was no significant difference in other characteristics. See Table 7.

**Table 7:** Characteristics of asphyxiated neonates that develop HIE within the first 24 h of admission to NCU

Variable	At admission				At 12 h				At 24 h			
	Total *n* = 282(%)	HIE Score		*p*-value	Total *n* = 243(%)	HIE Score		*P*-value	Total *n* = 244(%)	HIE Score		*P*-value
		1–10 *n* = 87(%)	> 10 *n* = 195(%)			1–10 *n* = 145(%)	> 10n = 119(%)			1–10 *n* = 184(%)	> 10 *n* = 60(%)	
New-born Sex				0.895				0.512				0.720
Female	128(45.4)	40(46.0)	88(45.1)		119(45.1)	68(46.9)	51(42.9)		109(44.7)	81(44.0)	28(46.7)	
Male	154(54.6)	47(54.0)	107(54.9)		145(54.9)	77(53.1)	68(57.1)		135(55.3)	103(56.0)	32(53.3)	
Gestational age (weeks)				0.012				0.041				0.417
< 37	22(7.8)	12(13.8)	10(5.1)		21(8.0)	16(11.0)	5(4.2)		18(7.4)	15(8.2)	3(5.0)	
37+	260(92.2)	75(86.2)	185(94.9)		243(92.0)	129(89.0)	114(95.8)		226(92.6)	169(91.8)	57(95.0)	
Color of the liquor				0.001				0.000				0.001
Clear	122(43.3)	54(62.1)	68(34.9)		119(45.1)	87(60.0)	32(26.9)		110(45.1)	96(52.2)	14(23.3)	
Meconium stained	160(56.7)	33(37.9)	127(65.1)		145(54.9)	58(40.0)	87(73.1)		134(54.9)	88(47.8)	46(76.7)	
Fetal presentation				0.073				0.465				0.494
Breech	26(9.2)	4(4.6)	22(11.3)		25(9.5)	12(8.3)	13(10.9)		23(9.4)	16(8.7)	7(11.7)	
Vertex	256(90.8)	83(95.4)	173(88.7)		239(90.5)	133(91.7)	106(89.1)		221(90.6)	168(91.3)	53(88.3)	
Newborn referred from outside facilities				0.002				0.002				0.001
No	179(63.5)	67(77.0)	112(57.4)		171(64.8)	106(73.1)	65(54.6)		163(66.8)	135(73.4)	28(46.7)	
Yes	103(36.5)	20(23.0)	83(42.6)		93(35.2)	39(26.9)	54(45.4)		81(33.2)	49(26.6)	32(53.3)	
Newborn been resuscitated				0.001				0.028				0.094
No	21(7.4)	15(17.2)	6(3.1)		22(8.3)	17(11.7)	5(4.2)		21(8.6)	19(10.3)	2(3.3)	
Yes	261(92.6)	72(82.8)	189(96.9)		242(91.7)	128(88.3)	114(95.8)		223(91.4)	165(89.7)	58(96.7)	
Newborn been initiated to breastfeeding				0.037				0.056				0.178
No	250(88.7)	72(82.8)	178(91.3)		233(88.3)	123(84.8)	110(92.4)		216(88.5)	160(87.0)	56(93.3)	
Yes	32(11.3)	15(17.2)	17(8.7)		31(11.7)	22(15.2)	9(7.6)		28(11.5)	24(13.0)	4(6.7)	
Newborn received any fluids resuscitation				0.009				0.202				0.043
No	113(40.1)	25(28.7)	88(45.1)		102(38.6)	51(35.2)	51(42.9)		95(38.9)	65(35.3)	30(50.0)	
Yes	169(59.9)	62(71.3)	107(54.9)		162(61.4)	94(64.8)	68(57.1)		149(61.1)	119(64.7)	30(50.0)	

### Multivariate analysis

Table 8 presents the multivariate analysis of factors associated with the development of Hypoxic-Ischemic Encephalopathy (HIE) in asphyxiated neonates admitted to the Neonatal Care Unit (NCU). Of all the factors included, three were found to be significantly associated with the development of HIE: meconium-stained amniotic fluid, referral of the newborn from outside facilities, and resuscitation of the newborn. Newborns with meconium-stained amniotic fluid had 2.2 times the likelihood of developing HIE compared to those with clear fluid [AOR, 2.2; 95% CI (1.2, 4.1); *P* = 0.014]. Newborns referred from outside facilities had 2.8 times the likelihood of developing HIE compared to those who were not referred [AOR, 2.8; 95% CI (1.4, 5.5); *P* = 0.002]. Finally, newborns who required resuscitation had 5.1 times the likelihood of developing HIE compared to those who were not resuscitated [AOR, 5.1; 95% CI (1.8, 15.0); *P* = 0.003]. See Table 8.

**Table 8:** Factors associated with the development of HIE at admission among newborns admitted at NCU, MRRH

Variable	COR (95% CI)	*P*-value	AOR (95% CI)	*P*-value
Mother’s age (age in years)				
15–19	1		1	
20–34	0.6(0.3, 1.1)	0.117	1.0(0.5, 2.2)	0.977
> 34	0.2(0.1, 0.7)	0.005	0.4(0.1, 1.2)	0.087
Delivered before term				
No	1		1	
Yes	0.4(0.2, 0.8)	0.016	0.6(0.2, 1.7)	0.370
Hypertension in pregnancy				
No	1		1	
Yes	0.4(0.2, 0.7)	0.003	0.6(0.2, 1.4)	0.257
Labor Obstructed				
No	1		1	
Yes	2.6(1.6, 4.5)	0.001	1.4(0.7, 2.6)	0.357
Gestational age (weeks)				
< 37	1		1	
37+	3.0(1.2, 7.1)	0.016	2.1(0.7, 6.2)	0.169
Colour of the meconium-stained amniotic fluid				
Clear	1		1	
Stained	3.1(1.8, 5.2)	0.016	2.2(1.2, 4.1)	**0.014**
Fetal presentation				
Vertex	1		1	
Breech	2.6(0.9, 7.9)	0.083	1.5(0.5, 5.1)	0.481
Newborn referred from outside facilities				
No	1		1	
Yes	2.5(1.4, 4.4)	0.002	2.8(1.4, 5.5)	**0.002**
Newborn been resuscitated				
No	1		1	
Yes	6.6(2.5, 17.6)	0.001	5.1(1.8, 15.0)	**0.003**
Newborns been initiated into breastfeeding				
No	1		1	
Yes	0.5(0.2, 0.9)	0.041	0.4(0.2, 1.0)	0.054
Newborn received any fluids resuscitation				
No	1		1	
Yes	0.5(0.3, 0.8)	0.010	0.8(0.4, 1.6)	0.497

## Discussions

In this study, we assessed factors that influence the development of (HIE) as an outcome in newborns with birth asphyxia. The factors described in this study included maternal demographic factors, intrapartum factors, newborn care, and newborn characteristics.

Despite recent advances in perinatal care, hypoxic-ischemic encephalopathy remains one of the most common causes of neonatal morbidity and mortality worldwide [21]. It refers to the various degrees of brain injury due to suffocation caused by partial or total lack of oxygen, reduction or suspension of the cerebral blood flow during the perinatal period [22]. Our study revealed a high incidence of hypoxic-ischemic encephalopathy that declined slightly over time from admission time at 70.3%,12 h at 45.1%% and 24.6%% at 24 h, respectively.

We found that meconium-stained amniotic fluid was associated with HIE. Newborns who had stained amniotic fluid were at higher risk of developing HIE compared to those born with clear amniotic fluid. The likely justification could be because meconium-stained amniotic fluid may be due to intrapartum inhalation, which leads to mechanical obstruction of airways, surfactant inactivation, chemical inflammation and apoptosis of the pulmonary tissues, thus facilitating pulmonary air leak and hypoxia [23]. This concurs with a case-control study that took place in Trousseau Hospital in Paris, which reported that 2.9% developed HIE [24, 25]and [26]. In terms of clinical significance, the association between meconium-stained amniotic fluid and hypoxic-ischemic encephalopathy underscores the importance of careful monitoring during labour. The presence of meconium not only indicates potential fetal distress but also heightens the risk of complications such as HIE.

Newborns who developed HIE were significantly more likely to have been referred from peripheral health facilities, undergone resuscitation with a bag and mask, and experienced delayed breastfeeding. These findings highlight potential gaps in timely intervention, which may be influenced by challenges such as an inadequate referral system, poor road infrastructure, and limited transportation options. Given the well-established association between birth asphyxia and neonatal outcomes, ensuring timely and high-quality perinatal care remains crucial. Mbale Regional Referral Hospital is the only facility in the region offering tertiary neonatal care, emphasizing the need to strengthen referral pathways and emergency obstetric services. Additionally, we also found that the use of a bag and mask to resuscitate newborns was significantly associated with HIE in our study. However, this association likely reflects the greater need for resuscitation among newborns with more severe intrapartum hypoxia-ischemia, rather than bag-mask ventilation itself contributing to HIE. The high incidence of HIE among resuscitated newborns may indicate delays in recognizing and managing birth asphyxia, as well as challenges in neonatal resuscitation, including limited access to equipment, trained personnel, and timely referrals. This is however contrary to the findings of Shikuku and friends who showed 90% improvement in newborns after resuscitation using a bag and mask [27]. Similarly, a study done in northern Uganda by Ayebare and others also used bag and mask ventilation for severely asphyxiated babies [28]. This finding implies that there is still a need to improve the referral systems of newborns that need care at neonate referral units and increase training in newborn resuscitation and care if the incidence of poor HIE is to be reduced.

We found that babies born to mothers aged 20–34 years were less likely to develop HIE than those born to mothers aged 15–19 years and that preterm infants were less likely to develop HIE than term infants. However, these differences were not statistically significant. Similarly, hypertension in pregnancy, obstructed labour, and fetal presentation were not found to be significant risk factors for HIE, despite being more commonly observed in mothers of affected newborns. The reasons for the variation in findings compared to other studies could not be explained.

### Strengths and limitations of the study

The strength of this study lies in its longitudinal design, tracking newborns for 24 h post-birth, and its comprehensive analysis of risk factors for birth asphyxia, including unique factors like referral from peripheral facilities and prior resuscitation. Conducted in a large public teaching and referral hospital with a diverse socio-economic catchment population, the study achieved its calculated sample size, enhancing the generalizability of findings and their relevance for policymaking. Efforts were made to ensure data reliability, although some limitations were acknowledged.

However, our study had several limitations. Data collection relied on interviewer-administered questionnaires and record abstraction, which may have introduced biases such as social desirability and acquiescence bias, though efforts were made to mitigate these through participant guidance. Congenital anomalies affecting breathing could not be assessed, nor could maternal obstetric history, birth events, and transportation details, which might have influenced newborn outcomes.

As a hospital-based study conducted in a regional referral facility with NICU access, findings may not fully reflect risk factors in community settings where many births lack access to such care. Additionally, the analysis used binary logistic regression for longitudinal outcomes, which, while effective, could be expanded upon in future studies using mixed-effects logistic regression for greater precision.

Lastly, the Kish Leslie Formula was not the ideal method for sample size calculation in a prospective study, though the data collected remained adequate and relevant. The focus on a population predominantly from low- to middle-income backgrounds further limits the generalizability to the wider national context.

## Conclusions

We identified significant associations between HIE and meconium-stained amniotic fluid, referrals from peripheral facilities, resuscitation using a bag and mask, and delays in initiating breastfeeding. These findings underscore the importance of addressing these risk factors during labour and the immediate postnatal period to reduce the incidence of HIE potentially. Optimising emergency responses and strengthening maternal and newborn care systems could play a pivotal role in mitigating these risks.

Based on our findings, we think the Ministry of Health should promote antenatal follow-up, strengthen healthcare workers’ skills, involve doctors in referral decisions, establish fully equipped ambulances at district health centers, and continue community sensitization efforts to encourage pregnant mothers to attend ANC visits, opt for facility-based deliveries, and seek skilled delivery assistance to improve maternal and neonatal outcomes. Continue community sensitization efforts to encourage pregnant mothers to attend ANC visits, opt for facility-based deliveries, and seek skilled delivery assistance to improve maternal and neonatal outcomes.

## Electronic supplementary material

Below is the link to the electronic supplementary material.

Supplementary Material 1

Supplementary Material 2

## Data Availability

Data is provided within the manuscript or supplementary information files.

## Abbreviations

APGAR: Appearance Grimace, Activity, and Respiration
BA: Birth Asphyxia
EMNCCG: Essential Maternal Newborn Clinical Care Guidelines
HIE: Hypoxic Ischemic Encephalopathy
IRB: Institution Research Board
MOH: Ministry of Health
MRRH: Mbale Regional Referral Hospital
MUAC: Mid Upper Arm Circumference
NCU: Neonatal Care Unit
PPROM: Preterm Premature Rupture of Membranes
SDG: Sustainable Development Goal
COR: Crude Odds Ratio
AOR: Adjusted Odds Ratio
WHO: World Health Organization
