# Supplementary material for: Birth asphyxia outcomes and associated factors among newborns admitted to a tertiary hospital in Eastern Uganda: A prospective cohort study
Source: BMC Pregnancy Childbirth. 2025 Apr 24;25:487. doi: 10.1186/s12884-025-07603-2 (PMC12023549; doi:10.1186/s12884-025-07603-2)
Supplement: Supplementary file 2 — Supplementary Material 2 [file 12884_2025_7603_MOESM2_ESM.pdf]

Serial number____________

DATA COLLECTION TOOL

INSTRUCTION: TICK WHERE APPLICABLE

TABLE 1: SOCIO-DEMOGRAPHIC INFORMATION

A. Mother's information

SN	Variable	Response
1	How old are you? (age in years)	---------
2	What is your weight (Kgs)	-----------
3	What is your height (cm)	------------
4	What is your marital status	1= Married<br>2= Single<br>3= separated/Divorced<br>4= widowed<br>5) = Others specify<br>…………………
5	What is your highest level of education	1= Non formal<br>2= primary<br>3=Secondary<br>4= Tertiary
6	What is your employment status	1= Employed<br>2= Not employed
7	How many children have you delivered	

B: Intrapartum condition

1	Did you deliver twins	1= Yes<br>2= No
2	Did your membranes rupture before onset of labour?	1= Yes<br>2= No
3	Did you deliver before term?	1= Yes<br>2=No
4	What was the mode of delivery	1= SVD<br>2=C/S<br>3= Instrumental delivery
5	How many hours did you take in active labour	
6	Did you hypertension in pregnancy	1=Yes<br>2=No
7		
8	Was labour Obstructed	1= Yes<br>2= No
9	Did you have bleeding before delivery	1=Yes<br>2=No
10	Did you attend ANC	1=Yes<br>2=No
11	How many times did you attend ANC	1=once<br>2= Two times<br>3=Three times<br>4=Four times<br>Others specify……………..
12	Where did you delivery from?	1= Hospital<br>2= At home<br>3= On my way to hospital

C: Characteristics and condition of Newborn at admission

1	New-born Sex	1) Male<br>2) Female
2	Birth weight (Kgs)	…..
3	Gestational age (weeks)	
4	What was the foetal presentation	1= Vertex<br>2= Breech
5	What was the colour of the liquor	1= Clear<br>2=Meconium stained
8		
9		

Immediate newborn care at admission

1	Is the new born referred from outside facilities	1= Yes<br>2= No
2	Has the new born been resuscitated	1= Yes<br>2= No
3	Has the new born been initiated to breastfeeding	1= Yes<br>2= No
4	What is the new born temperature at admission(oc)	
5	Has the new born received any fluids resuscitation	1= yes<br>2= no

D: **Immediate outcomes of newborn with birth asphyxia** at admission

1	Has the baby developed HIE	1=Yes<br>2= No

2	What is the score of HIE	1= 0-10: HIE Mild<br><br>2= 11-14: HIE Moderate<br><br>3= ≥15: HIE Severe
3	What is the new-borns SPO2 (%)	
4	How many hours has the new born spent in NICU<br>From time of admission?	
5	Has the new born been discharged	1=yes<br><br>2=No
6	Has the new-born died	1= yes<br><br>2= No

Immediate outcomes of newborn with birth asphyxia at 12hours

1	Has the baby developed HIE	1=Yes<br><br>2= No
2	What is the score of HIE	1= 0-10: HIE Mild<br><br>2= 11-14: HIE Moderate<br><br>3= ≥15: HIE Severe
3	What is the new-borns SPO2 (%)	
4	How many hours has the new born spent in NICU<br>From time of admission?	

5	Has the new born been discharged	1=yes<br><br>2=No
6	Has the new-born died	1= yes<br><br>2= No

Immediate outcomes of newborn with birth asphyxia at 24 hours

1	Has the baby developed HIE	1=Yes<br><br>2= No
2	What is the score of HIE	1= 0-10: HIE Mild<br><br>2= 11-14: HIE Moderate<br><br>3= ≥15: HIE Severe
3	What is the new-borns SPO2 (%)	
4	How many hours has the new born spent in NICU<br>From time of admission?	
5	Has the new born been discharged	1=yes<br><br>2=No
6	Has the new-born died	1= yes<br><br>2= No

Appendix VI: Tool

Thompson Score for Hypoxic Ischemic Encephalopathy

Pleases use this form to score all live babies at admission , 12 hours and 24hours after delivery

**Sign**	**0**	**1**	**2**	**3**
Tone	Normal	Hyper	Hypo	Flaccid
LOC (level of consciousness)	Normal	Hyper alert, stare	Lethargic	Comatose
Fits	None	<3 per day	>2 per day	
Posture	Normal	Fisting/cycling	Strong distal flexion	Decelerate
Moro reflex	Normal	Partial	Absent	
Grasp	Normal	Poor	Absent	
Sucking reflex	Normal	Poor	absent± bites	
Respiration	Normal	hyperventilation	brief apnea	Intermittent Positive Pressure Ventilation (apnoea)
Fontanel	Normal	Full, not tense	Tense	

Indicate the assessment scores in the table below

**Date**				
**Time(hours)**				
Tone				
LOC				
Fits				
Posture				
Moro				
Grasp				
Suck				
Respiration				
Fontanel				
**TOTAL Score**				

Assessed by ____

Key

The final score sums the individual points

0-10: HIE Mild

11-14: HIE Moderate

≥15: HIE Severe
